# Comparative analysis of intestinal morphology and intestinal microbiota composition of bullfrogs (*Aquarana catesbeiana*) at different growth stages

**DOI:** 10.3389/fmicb.2025.1715163

**Published:** 2026-01-05

**Authors:** Jingyi Xie, Xiaoting Zheng, Qiuyu Chen, Xueying Liang, Hongbiao Dong, Shengfu Zhou, Xiaoquan Yuan, Jiasong Zhang

**Affiliations:** 1 College of Fisheries and Life Science, Shanghai Ocean University, Shanghai, China; 2 Key Laboratory of South China Sea Fishery Resources Exploitation and Utilization, South China Sea Fisheries Research Institute, Ministry of Agriculture and Rural Affairs, Chinese Academy of Fishery Sciences, Guangzhou, China; 3 Key Laboratory of Efficient Utilization and Processing of Marine Fishery Resources of Hainan Province, Sanya Tropical Fisheries Research Institute, Lingshui, China; 4 Fuzhou Agricultural Sciences Research Institute, Fuzhou, Jiangxi, China

**Keywords:** bullfrog, intestinal morphology, 16S rRNA, intestinal microbiota, symbiotic probiotics

## Abstract

The intestinal microbiota is a complex and dynamic community that contributes to digestion and plays a crucial role in regulating immune health. In this study, post-metamorphic bullfrogs (*Aquarana catesbeiana*) at different ages (1, 2, 3, and 4 months) were investigated. Growth performance assessment, intestinal histomorphological analysis, and 16S rRNA sequencing were employed to systematically examine the dynamics and diversity of microbial communities in the small intestinal segments (duodenum, jejunum, and ileum). Results showed that bullfrog growth indices increased with age, with faster body weight gain during 2–3 months; notably, this was significantly positively correlated with intestinal morphological development (villus height and muscle layer thickness) (*p* < 0.05). In terms of microbial composition, Firmicutes, Proteobacteria, Bacteroidetes, Fusobacteria, and Actinobacteria were dominant phyla, while different intestinal segments harbored specific dominant genera. Among them, *Cetobacterium* was consistently detected throughout the growth period, suggesting it is likely the core symbiont in bullfrog intestines. Moreover, microbiota function varied with growth stages: at 1–2 months, *Bifidobacterium* and *Cetobacterium* synergistically participated in immune regulation and basic metabolism, whereas at 3–4 months, *Weissella*, *Lactococcus*, and *Bacteroides* became dominant, with their functions shifting toward efficient energy conversion. Additionally, Alpha diversity analysis showed a decreasing trend in the Simpson index with development, while Beta diversity analysis revealed that microbiota composition was similar among different intestinal segments at the same age but that significant differences existed in each segment during 2–3 months. Overall, this study reveals the specific distribution characteristics of probiotic microbiota in bullfrogs at different growth stages, thereby providing a scientific basis for screening growth-promoting frog-derived probiotics that match host physiological traits.

## 1 Introduction

Comprising tens of thousands of microorganisms, the intestinal microbiota forms a complex and dynamic ecosystem that partakes in food digestion and regulates both the host’s immune function and overall health (Kolodziejczyk et al., 2019). The stability of its community is collectively modulated by multiple factors, including host genetics, developmental stages, environmental conditions, and dietary compositions (Abdul Razak and Scribner, 2020). Intestinal physiological remodeling induced by growth and development is frequently accompanied by significant shifts in microbiota structure (Li et al., 2023). Amphibians, characterized by their distinct life history, represent an ideal model for investigating variations in intestinal microbial communities across different structural and developmental stages (Tong et al., 2020). During their development, the intestinal microbiota exhibits a unique evolutionary trajectory: transitioning from a fish-like microbial community in the tadpole stage to a structure more analogous to that of amniotes (including mammals, birds, and bipedal reptiles) (Kohl et al., 2013). However, the intestinal immune system of frogs is more primitive than that of mammals (Ruiz and Robert, 2023), meaning microbiota succession may rely more on the “microenvironmental adaptability” provided by intestinal morphological development. The synchronous association between these two processes remains unclear.

The bullfrog (*Aquarana catesbeiana*) has gradually emerged as one of the major species in aquaculture due to its rapid growth and high yield (Weng et al., 2025). However, its characteristic rapid growth within 4 months post-metamorphosis has led to the widespread adoption of high-density feeding regimes in intensive farming systems (Ye et al., 2012). This, in turn, has triggered issues such as antibiotic overuse and microecological imbalance (Zhang H., 2023; Zhang Z. Y., 2023), highlighting an urgent need for targeted microecological regulation strategies. Existing studies on bullfrog intestinal microbiota still have notable gaps. Temporally, most focus on the metamorphic stage (tadpole-to-juvenile transition) or adulthood, with no tracking of microbiota dynamics during the critical 1–4-month growth period. Spatially, research remains oversimplified: most use the entire intestine as the unit, without subdividing into the duodenum, jejunum, and ileum—preventing the revelation of how segment-specific functional differences screen microbiota distribution.

In this study, 16S rRNA sequencing technology was employed to systematically analyze the microbial community structure and its dynamic changes in the duodenum, jejunum, and ileum of bullfrogs at different post-metamorphic ages (1–4 months), aiming to reveal the spatiotemporal evolution patterns of their intestinal microbiome. By integrating the growth requirements of each stage, we screened frog-derived probiotics with host adaptability, which could provide a theoretical basis for the development of microecological agents in healthy bullfrog farming and facilitate the green, efficient, and sustainable development of aquaculture.

## 2 Materials and methods

### 2.1 Animals

The bullfrogs (*Aquarana catesbeiana*) were obtained from Guangzhou Shengshi Tangfeng Aquaculture Farm (23.4390°N, 113.2503°E). Embryos were obtained from sexually mature frogs via natural oviposition in March and allowed to hatch in nylon mesh tanks (80 cm × 80 cm × 70 cm, mesh size: 80 μm) with 30 cm of water (without any manipulation during hatching), and subsequently maintained in a recirculating aquaculture system in plastic tanks (3 m × 5 m × 0.6 m, water depth: 0.3 m). During the entire rearing period, the bullfrogs were fed Skretting compound feed for bullfrogs. Regular sampling was conducted once a month for a total of four times, corresponding to 1, 2, 3, and 4 months post-metamorphosis, which were recorded as groups D, E, F, and G, respectively. At each sampling, 10 bullfrogs were randomly selected from the rearing tanks, and indicators such as body weight, body length, and head length were measured. Six bullfrogs per group were euthanized by spinal cord transection. From each frog, 1 cm segments of the duodenum, jejunum, and ileum were isolated, and an appropriate amount of intestinal contents (more than 0.2 g) were scraped, placed into 2 mL cryopreservation tubes, homogenized, rapidly frozen in liquid nitrogen, and stored in a −80 °C refrigerator for subsequent intestinal microbiota analysis. Meanwhile, 5 mm tissue segments from the remaining duodenum, jejunum, and ileum were fixed in 4% paraformaldehyde solution and stored in 5 mL cryopreservation tubes for tissue section preparation.

### 2.2 16S rRNA gene sequencing

The extraction and detection of bacterial DNA, PCR amplification, and NovaSeq sequencing were carried out by Microbial Group Co., Ltd. in Shenzhen, China. The experimental procedure is as follows: First, the total genomic DNA was extracted from the intestinal contents samples using the CTAB method. Next, the V3-V4 variable regions were subjected to PCR amplification with the fusion primers 341F (5′-CCTAYGGGRBGCASCAG-3′) and 806R (5′-GGACTACNNGGGTATCTAAT-3′) with barcodes. Then, the DADA2 plugin in the QIIME2 software was used to perform quality control, denoising, splicing, and chimera removal on all original sequences of all samples to form Operational Taxonomic Units (OTUs). Finally, the representative sequences of the OTUs were selected and compared against the Silva database (default: Silva Release 132 for 16S rRNA genes) to obtain species annotation information (Ceccarani and Severgnini, 2023). Various methods, including ANCOM, Kruskal-Wallis, ANOVA, LEfSe, DESeq2, etc., were employed to analyze the significant differences in the taxonomic levels of species among groups. All data analyses was processed using the online platform WeKemo BioCloud.

### 2.3 Intestinal histological processing

The intestinal tissues were preserved in a 4% paraformaldehyde solution and then stained with hematoxylin–eosin (HE staining). The sections were scanned using a PANNORAMIC panoramic slide scanner, and then imaged using CaseViewer 2.4. After imaging was completed, the Image-Pro Plus 6.0 software was used to measure the intestinal epithelial height and the thickness of the muscular layer. The HE sections and light microscopy images were prepared by Wuhan ServiceBio Biotechnology Co., Ltd.

### 2.4 Statistical analysis

Experimental data were processed using Excel 2019, and then one-way analysis of variance (ANOVA) and Duncan’s multiple range test were performed using SPSS 22.0 to analyze the significant differences among groups across different developmental stages. *p* < 0.05 was considered statistically significant, and the data were expressed as “mean ± standard error (mean ± SE).” Statistically significant differences were visualized using GraphPad Prism 9 software.

## 3 Results

### 3.1 Rapid morphological growth of bullfrogs from 1 to 4 months post-metamorphosis

**Figure 1:**
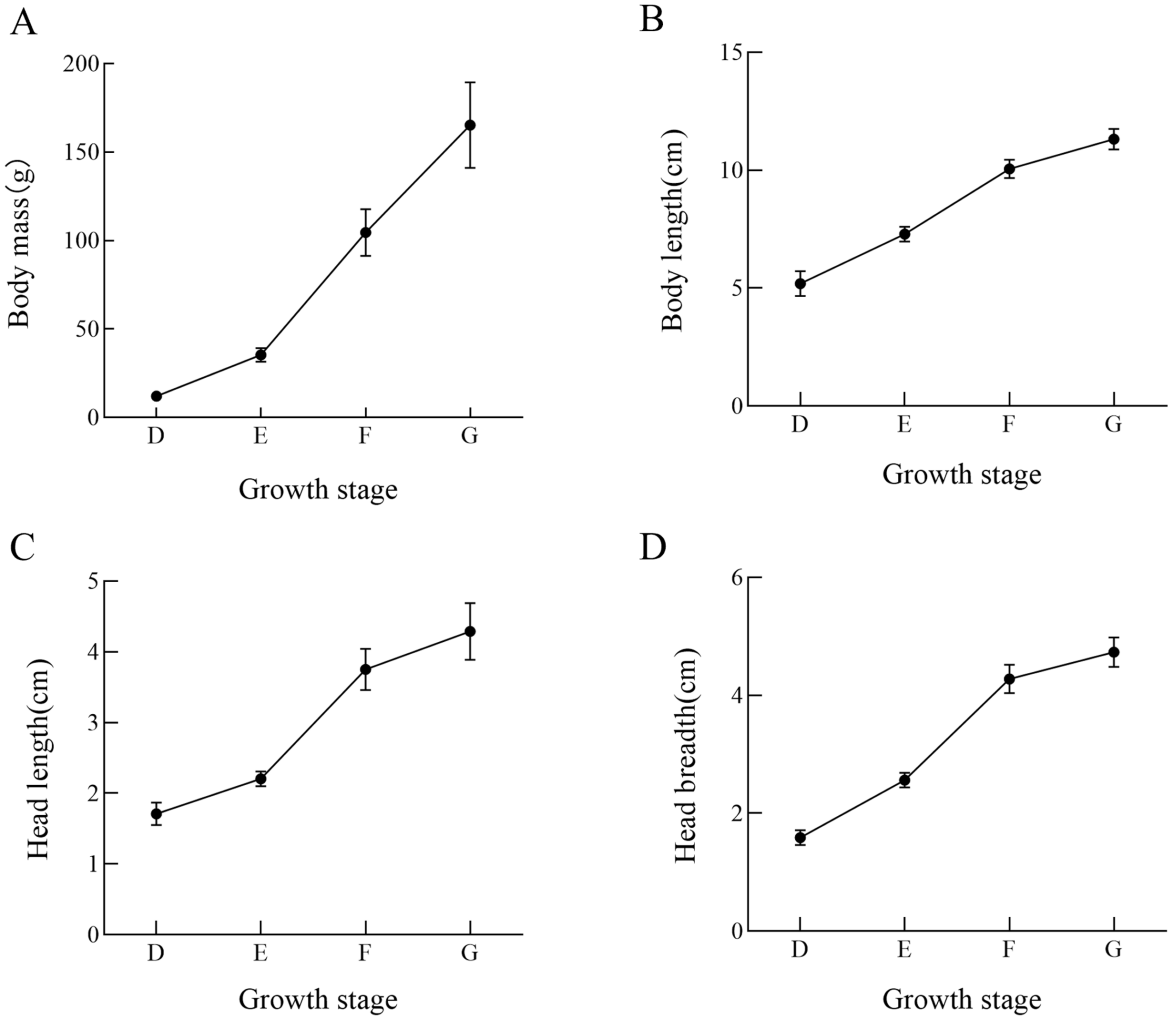
Morphological measurements of bullfrogs at four different growth stages (the first month, the second month, the third month and the fourth month after bullfrog metamorphosis). **(A)** Body mass **(B)** body length **(C)** head width **(D)** head length. *n* = 10 bullfrogs per developmental stage.

Bullfrogs showed a continuous growth trend in morphological traits including body weight, body length, head length, and head breadth from 1 to 4 months post-metamorphosis (Table 1; Figure 1). Specifically, from the 2nd to the 3rd post-metamorphosis, their body weight, head length, and head breadth increased rapidly, with growth rates of 233, 74, and 67%, respectively.

**Table 1:** Growth indexes of bullfrog at different stages (Mean ± SEM, *n* = 10).

Group	Body weight (g)	Body length (cm)	Head length (cm)	Head breadth (cm)
D	12.13 ± 1.32^a^	5.20 ± 0.53^a^	1.71 ± 0.16^a^	1.59 ± 0.13^a^
E	35.44 ± 4.36^b^	7.29 ± 0.34^b^	2.19 ± 0.11^b^	2.56 ± 0.14^b^
F	117.92 ± 23.32^c^	10.17 ± 0.45^c^	3.8 ± 0.32^c^	4.28 ± 0.26^c^
G	168.19 ± 26.88^d^	11.32 ± 0.47^d^	4.2 ± 0.45^d^	4.73 ± 0.28^d^

The data in the table are the average of 10 replicates; groups D, E, F, and G correspond to bullfrogs 1 month old, 2 months old, 3 months old, and 4 months old after metamorphosis, respectively.

### 3.2 Age-related changes in bullfrog intestinal morphology

**Figure 2:**
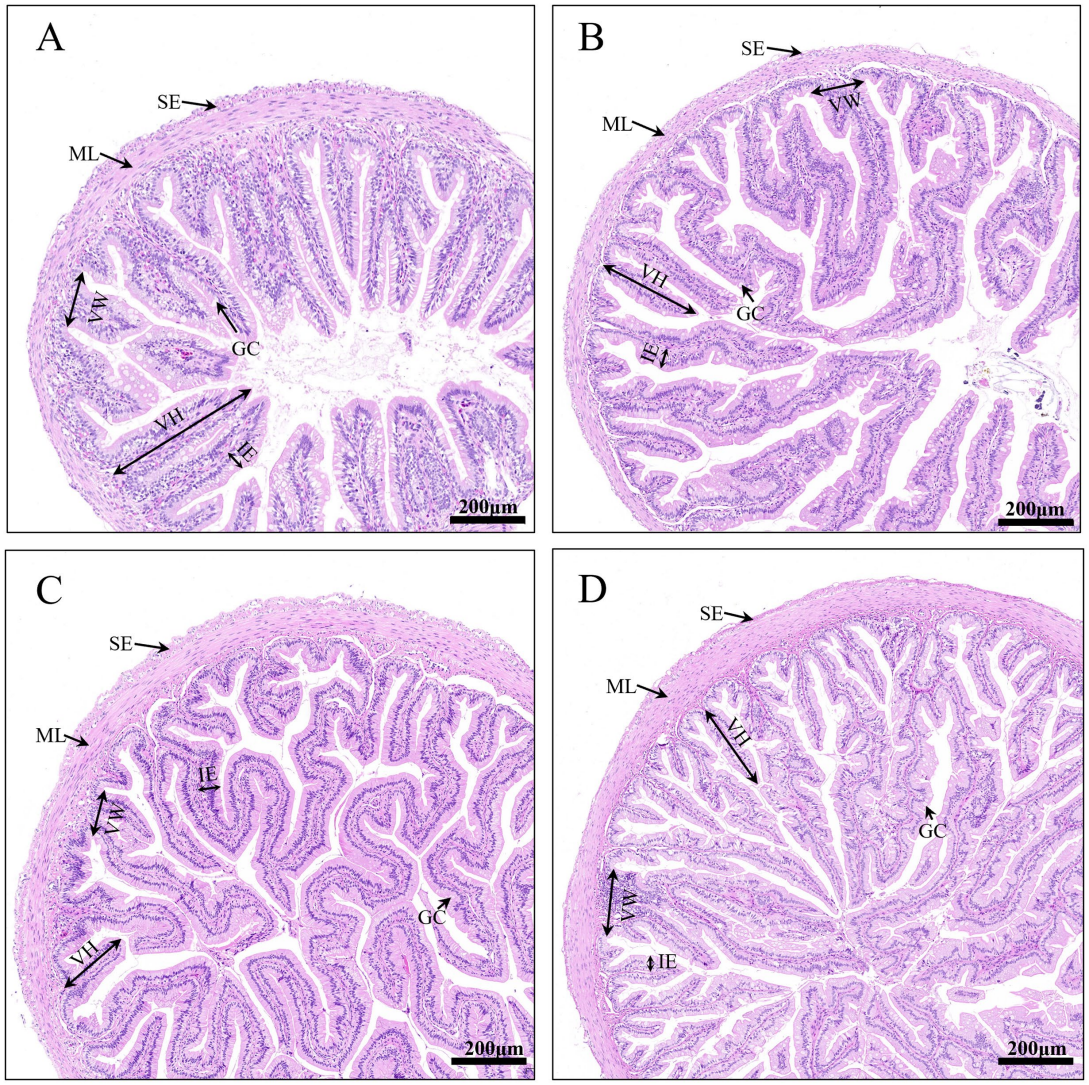
Intestinal morphology of bullfrog at different stages. **(A)** Group D (1 month post-metamorphosis), **(B)** Group E (2 months post-metamorphosis), **(C)** Group F (3 months post-metamorphosis), **(D)** Group G (4 months post-metamorphosis). The a–d scale is 200 μm. ML, Muscular layer; SE, serosa; GC, goblet cells; VH, villus height; VW, villus width; IE, intestinal epithelial height.

Significant differences were observed in intestinal villus height and intestinal muscular layer thickness among different stages (Table 2). Intestinal villus height gradually increased with increasing age, and the villi became increasingly curled and dense (Figure 2). The intestinal villus height in group G was significantly higher than that in group D (*p* < 0.05); the intestinal muscular layer thickness in both groups F and G was significantly higher than that in groups D and E (*p* < 0.05). No significant differences were observed in intestinal villus width and intestinal epithelial height (*p* > 0.05).

**Table 2:** Intestinal structure parameters of bullfrog across developmental stages (Mean ± SEM, *n* = 3).

Parameters (μm)	D	E	F	G
Villus height	700.95 ± 201.36^a^	771.17 ± 106.19^ab^	791.81 ± 141.77^ab^	1069.62 ± 250.47^b^
Villus width	122.62 ± 15.78	119.45 ± 22.04	114.5 ± 12.35	116.20 ± 19.93
Muscular layer thickness	48.86 ± 15.92^a^	43.55 ± 11.10^a^	84.21 ± 24.26^b^	89.04 ± 8.83^b^
Intestinal epithelial height	52.89 ± 8.20	63.33 ± 2.87	55.55 ± 4.62	57.50 ± 7.19

Data are means of triplicates. Bars with different letters in the same row indicate significant differences (*P* < 0.05). Groups D, E, F, and G correspond to bullfrogs 1 month old, 2 months old, 3 months old, and 4 months old after metamorphosis, respectively.

### 3.3 Analysis of microbial microbiota in different intestinal segments of bullfrog at the same stage

#### 3.3.1 Age- and segment-dependent changes in bullfrog intestinal microbial composition

**Figure 3:**
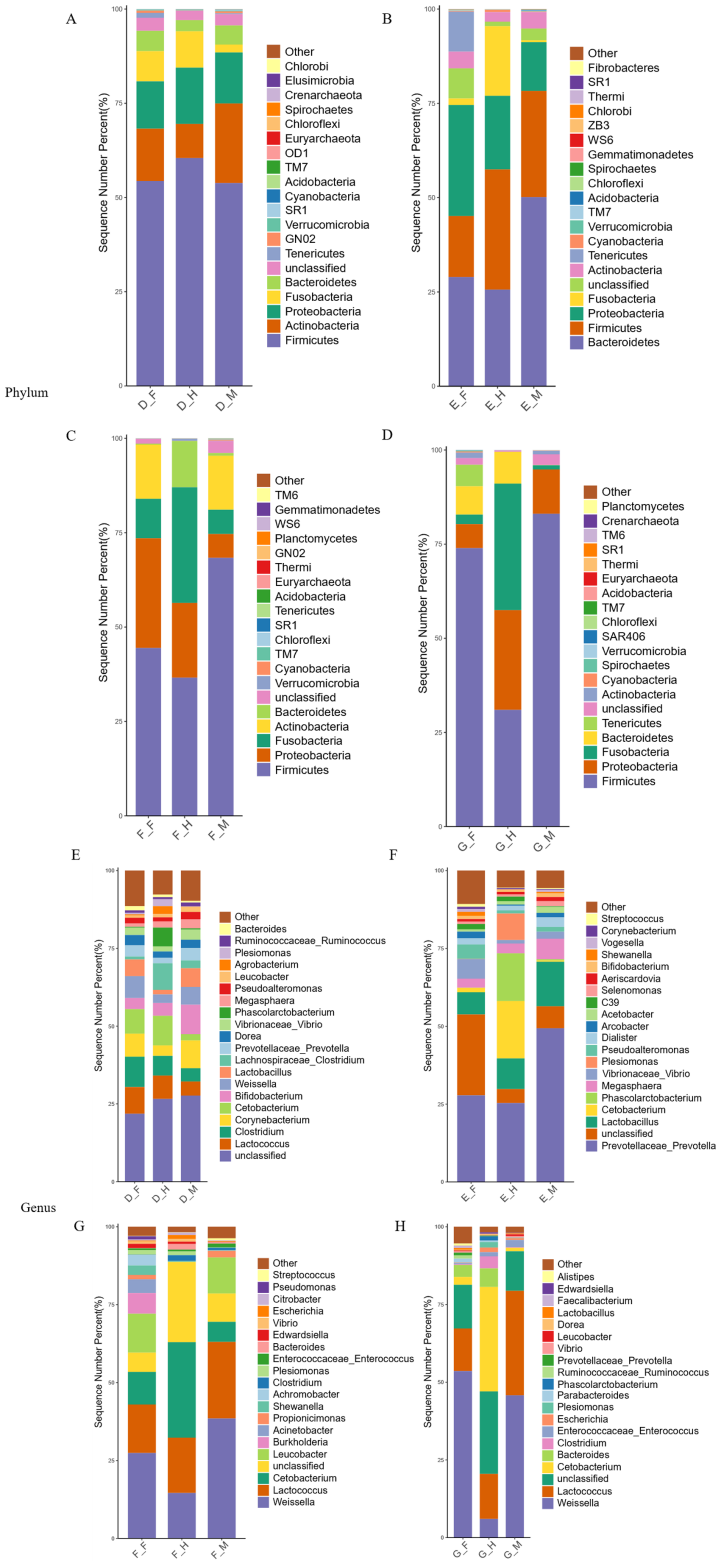
Histogram of relative distribution of phylum and genus levels in different intestinal segments of bullfrog at the same stage. **(A–D)** Bar chart of phylum-level relative abundance for Groups D–G. **(E–H)** Bar chart of genus-level relative abundance for Groups D–G. F stands for duodenum, M for jejunum, and H for ileum, the same applies below.

Phylum-level analysis of microbial composition across different intestinal segments in bullfrogs at the same growth stage (Figure 3), revealed that Firmicutes, Actinobacteria, Proteobacteria, Fusobacteria, and Bacteroidetes were the top five phyla in terms of relative abundance in the bullfrog intestine, collectively accounting for over 80% of the total microbial community.

At the genus level, the dominant bacterial genera varied across intestinal segments in Group D: *Clostridium* and *Lactococcus* predominated in the duodenum; *Corynebacterium* and *Bifidobacterium* were the dominant genera in the jejunum; while *Cetobacterium* and *Lactococcus* dominated the ileum. In Group E, *Prevotella* and *Lactobacillus* emerged as the dominant genera in both the duodenum and jejunum, with *Clostridium* and *Prevotella* being the primary dominant genera in the ileum. For Group F, *Weissella* and *Lactococcus* constituted the major bacterial genera in both the duodenum and jejunum, whereas *Lactococcus* and *Cetobacterium* dominated the ileum. In Group G, *Weissella* and *Lactococcus* were among the main dominant genera across the duodenum, jejunum, and ileum.

In the comparative analysis of specific genera abundance, Group D exhibited a gradual decrease in *Weissella* abundance from the duodenum to the ileum (ranging from 7 to 2%). *Lactococcus*, *Cetobacterium*, and *Clostridium* showed relatively high abundances in the duodenum and ileum, whereas *Corynebacterium* and *Bifidobacterium* were concentrated in the jejunum. *Phascolarctobacterium* reached 6% abundance in the ileum but only 0.3% in other segments. In Group E, *Prevotella* and *Lactobacillus* were most abundant in the jejunum. Notably, *Phascolarctobacterium* in the ileum increased to 15%, significantly higher than the 0.04–0.3% observed in other segments, while *Vibrio* was relatively enriched in the duodenum. For Group F, *Weissella* and *Lactococcus* dominated the jejunum. *Cetobacterium* reached a high abundance of 30% in the ileum, compared to only 6–10% in the other two segments, and *Leucobacter* showed relatively higher levels in the duodenum and jejunum. In Group G, *Weissella* abundance decreased progressively along the intestinal tract (45–53% in the duodenum and jejunum vs. 6% in the ileum). *Lactococcus* was significantly enriched in the jejunum, and *Cetobacterium* was primarily distributed in the ileum.

**Figure 4:**
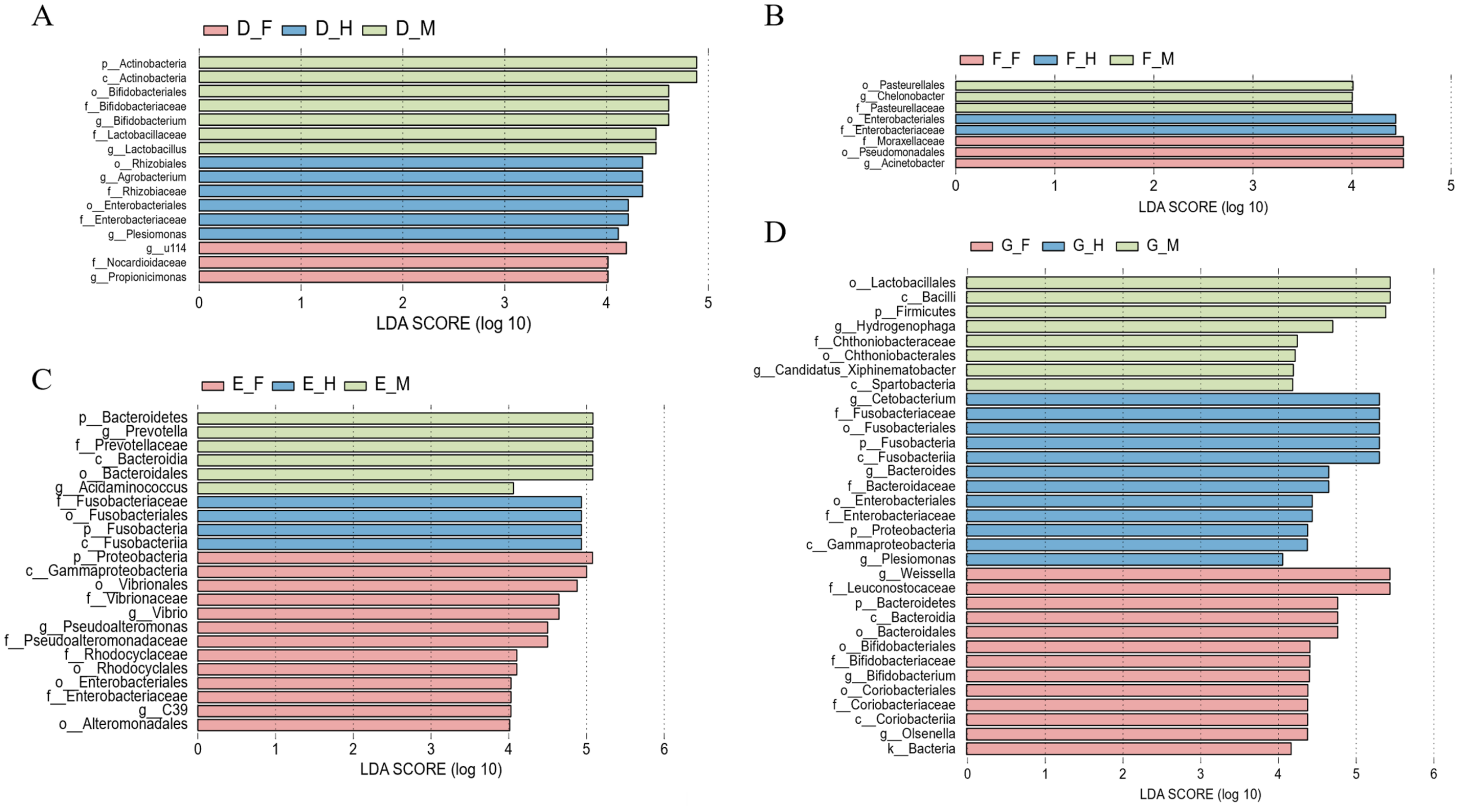
The LDA histogram of microbial genus levels in different intestinal segments of bullfrog at the same stage was analyzed by LEfSe, LDA > 4. **(A)** Group D (1 month post-metamorphosis); **(B)** group E (2 months post-metamorphosis); **(C)** group F (3 months post-metamorphosis); **(D)** group G (4 months post-metamorphosis).

To identify the differentially dominant bacterial genera across intestinal segments, LEfSe (Linear Discriminant Analysis Effect Size) analysis was employed to screen for biomarkers with an LDA score > 4 and statistical significance (*p* < 0.05) (Figure 4). The results revealed that Group D contained 16 significantly different bacterial taxa (*p* < 0.05), among which 7 taxa (including *Bifidobacterium* and *Lactobacillus*) exhibited higher abundances in the jejunum. Group E harbored 23 significantly different bacterial taxa (*p* < 0.05), with 13 taxa (e.g., *Vibrio* and *Pseudoalteromonas*) showing elevated abundance in the duodenum. Group F presented 8 significantly differential bacterial taxa (*p* < 0.05), where the duodenum and jejunum each contained 3 dominant taxa, including *Acinetobacter*. Group G comprised 33 significantly differential bacterial taxa (*p* < 0.05), among which 13 taxa enriched in the duodenum included *Weissella* and *Bacteroides*.

**Figure 5:**
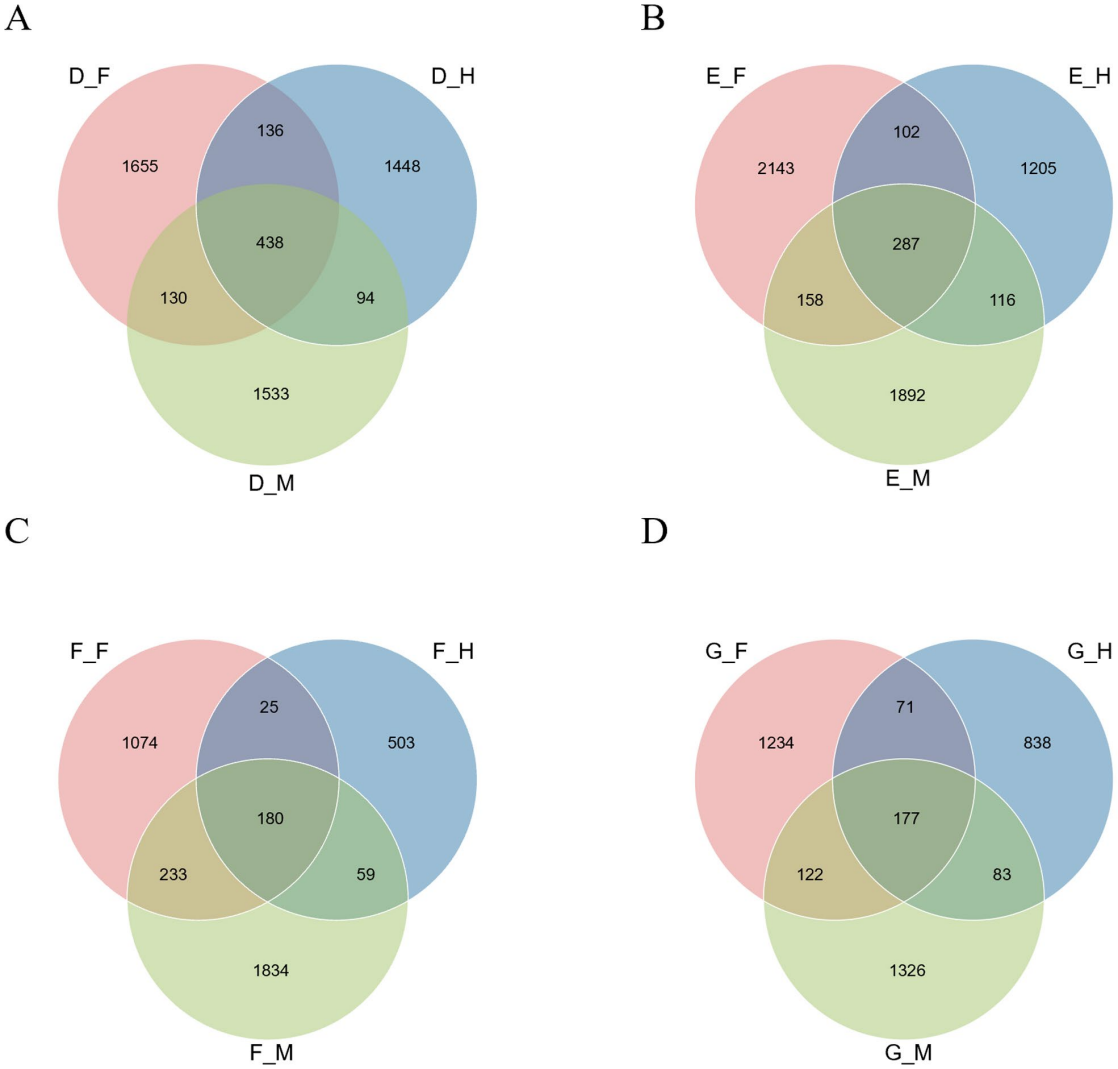
OTUs microorganisms in different intestinal segments of bullfrog at the same stage. **(A)** Group D (1 month post-metamorphosis). **(B)** Group E (2 months post-metamorphosis). **(C)** Group F (3 months post-metamorphosis). **(D)** Group G (4 months post-metamorphosis).

A further analysis of the microbial community characteristics across different intestinal segments at the same developmental stage (Figure 5) showed that Group D, Group E, Group F, and Group G contained 5,434, 5,903, 3,908, and 3,851 operational taxonomic units (OTUs), respectively. The number of OTUs shared among intestinal segments within each group decreased with increasing post-metamorphic age. In Group D, the number of OTUs shared across all intestinal segments was the highest, reaching 438; within this group, the ileum contained 1,448 unique OTUs, a number higher than that of unique OTUs in the corresponding segments of other groups. Group E had 287 OTUs shared across all intestinal segments; among its segments, the duodenum and jejunum possessed the highest number of unique OTUs compared to the other three groups, with 2,143 and 1892, respectively. Groups F and G exhibited fewer OTUs shared across all intestinal segments, with 180 and 177, respectively.

In summary, analyses of bullfrog intestinal microbiota across different intestinal segments at the same developmental stage (1–4 months post-metamorphosis) revealed that five phyla (Firmicutes, Actinobacteria, Proteobacteria, Fusobacteria, Bacteroidetes) dominated the community (>80% of total microbiota) at the phylum level, while at lower taxonomic levels (genus, OTU) and in functional biomarker screening (via LEfSe), distinct patterns emerged: dominant genera varied by intestinal segment and age, specific genera showed segment-specific enrichment (e.g., *Cetobacterium* in the ileum), differential biomarkers differed among age groups, and the number of OTUs shared across intestinal segments within each group decreased with increasing age.

#### 3.3.2 Age-dependent variations in microbial diversity across bullfrog intestinal segments

To assess differences in microbial community diversity among distinct intestinal segments of bullfrogs at the same developmental stage, the Wilcoxon rank-sum test was utilized to compare alpha diversity indices across intestinal segments (Table 3). In Group D, both the observed species index and Chao1 index exhibited a gradual decrease from the duodenum to the ileum, with no statistically significant differences detected (*p* > 0.05). For Group E, the observed species index, Chao1 index, and Shannon index all showed a progressive decline along the duodenum-to-ileum axis, and the Shannon index differed significantly between the duodenum and ileum (*p* < 0.05). In Group F, significant differences were observed in both the observed species index and Chao1 index between the jejunum and ileum (*p* < 0.05). In Group G, the Observed index and Chao1 index in the jejunum were significantly higher than those in the ileum; the Shannon index and Simpson index showed no significant differences among the three intestinal segments.

**Table 3:** Effects of microbial alpha diversity index in different intestinal segments of bullfrog at different stages.

Main group	Group	Observed index	Chao1 index	Shannon index	Simpson index
D	D_F	573.83 ± 181.57^a^	575.47 ± 182.59^a^	5.86 ± 1.35^a^	0.92 ± 0.08^ab^
	D_M	545.33 ± 47.61^ab^	547.24 ± 48.32^ab^	6.28 ± 0.40^a^	0.97 ± 0.01^a^
	D_H	530.00 ± 132.77^abc^	531.77 ± 132.08^abc^	5.50 ± 0.49^ab^	0.92 ± 0.04^ab^
E	E_F	578.50 ± 163.22^a^	579.45 ± 162.91^a^	5.95 ± 1.06^a^	0.91 ± 0.13^ab^
	E_M	558.17 ± 79.11^a^	560.09 ± 79.22^a^	5.38 ± 0.41^ab^	0.92 ± 0.02^ab^
	E_H	413.67 ± 118.31^abcde^	415.95 ± 118.07^abcde^	4.28 ± 1.56^bc^	0.81 ± 0.18
F	F_F	323.50 ± 162.97^bcde^	323.96 ± 163.15^bcde^	2.95 ± 0.24^c^	0.73 ± 0.08^c^
	F_M	491.67 ± 357.56^abcd^	492.84 ± 357.57^abcd^	3.24 ± 1.63^c^	0.69 ± 0.27^c^
	F_H	201.83 ± 66.76^e^	202.89 ± 67.91^e^	3.20 ± 0.52^c^	0.77 ± 0.05^bc^
G	G_F	311.50 ± 182.56^cde^	311.81 ± 182.75^cde^	3.37 ± 1.78^c^	0.74 ± 0.15^c^
	G_M	361.17 ± 253.02^abcde^	361.77 ± 252.69^abcde^	3.01 ± 0.72^c^	0.73 ± 0.07^c^
	G_H	265.00 ± 120.46^de^	266.23 ± 121.60^de^	3.20 ± 1.17^c^	0.74 ± 0.16^c^

F stands for duodenum, M for jejunum, and H for ileum. Data are means of triplicates. Bars with different letters in the same row indicate significant differences (*P* < 0.05). Groups D, E, F, and G correspond to bullfrogs 1 month old, 2 months old, 3 months old, and 4 months old after metamorphosis, respectively.

#### 3.3.3 Minimal beta diversity across intestinal segments in bullfrogs at the same developmental stage

**Figure 6:**
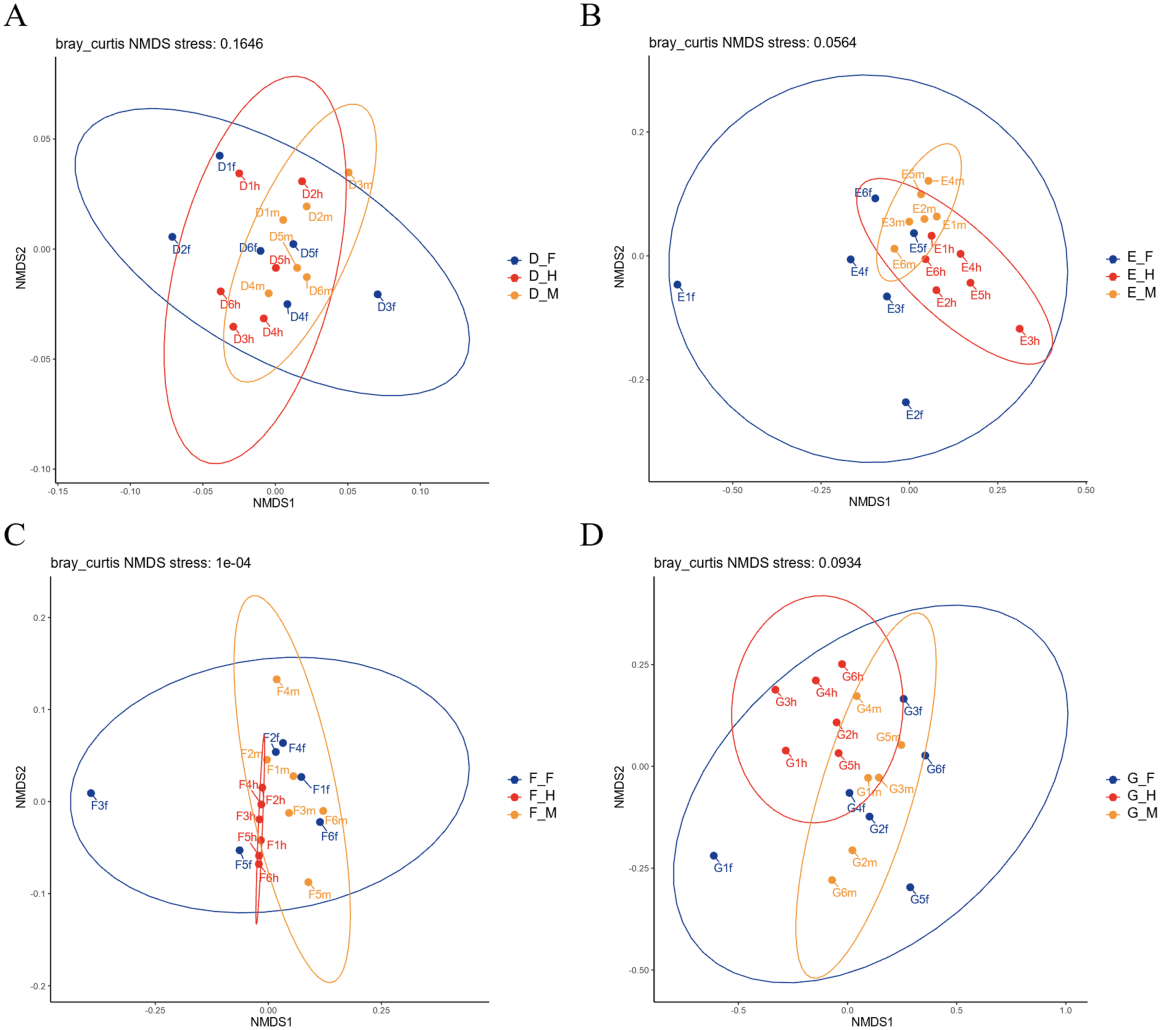
NMDS analysis of bullfrog in different intestinal segments at the same stage: **(A)** Group D (1 month post-metamorphosis); **(B)** Group E (2 months post-metamorphosis); **(C)** Group F (3 months post-metamorphosis); **(D)** Group G (4 months post-metamorphosis).

Beta diversity was analyzed using non-metric multidimensional scaling (NMDS) based on Bray-Curtis distances. A shorter distance between samples indicated greater similarity in species composition; consequently, samples with high community similarity clustered together, whereas those with distinct community compositions were separated. The results of beta diversity analysis among different intestinal segments of bullfrogs at the same developmental stage revealed that the community compositions in the duodenum, jejunum, and ileum were similar within each group (Figure 6).

### 3.4 Analysis of microbial microbiota in different stages of the same intestinal segment of bullfrog

#### 3.4.1 Developmental stage-dependent shifts in bullfrog intestinal microbiota composition

**Figure 7:**
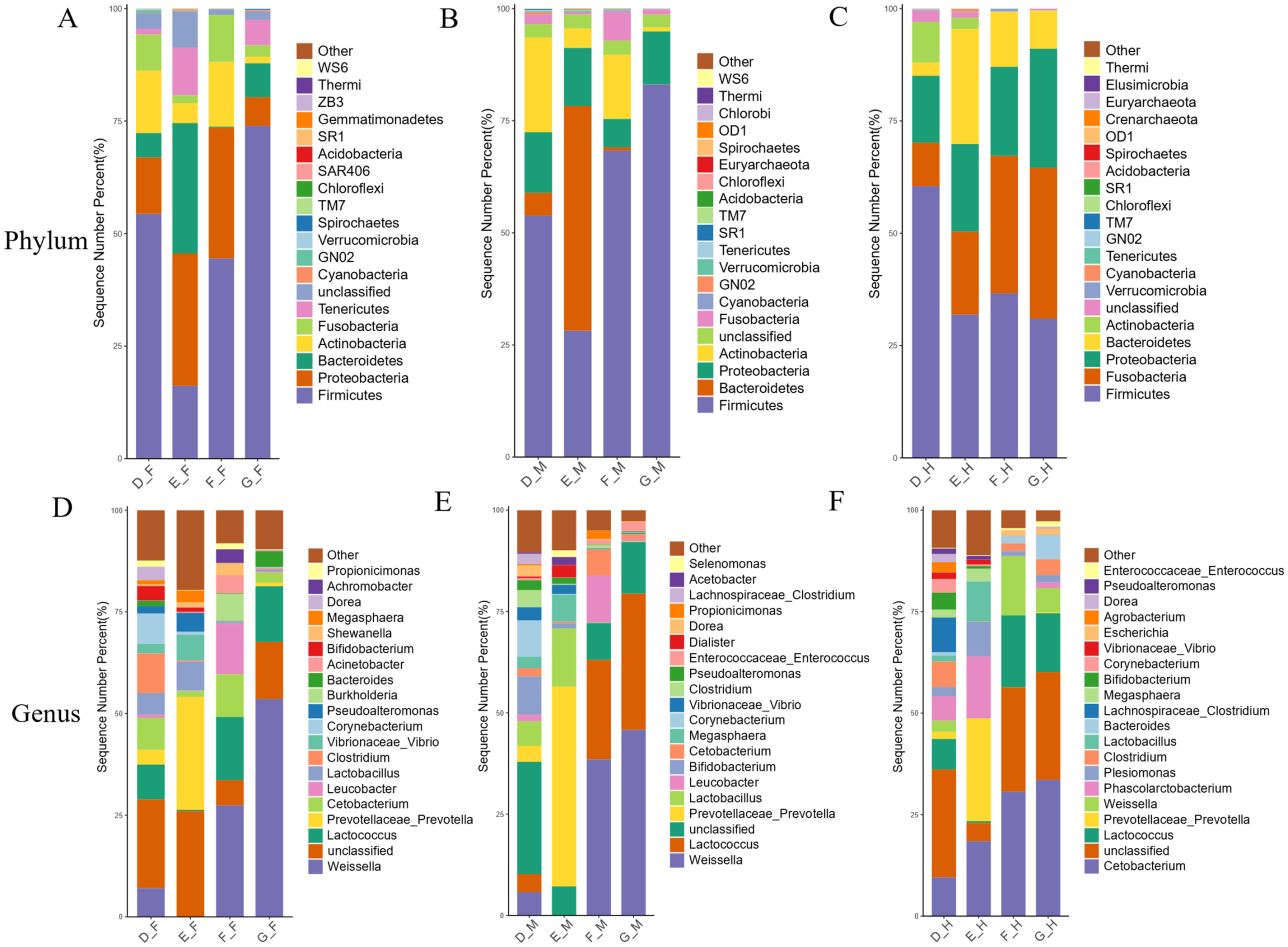
Histogram of relative distribution of phylum and genus levels in the same intestinal segment of bullfrog at different stages. **(A)** Bar chart of phylum-level relative abundance in duodenum across groups; **(B)** bar chart of phylum-level relative abundance in jejunum across groups; **(C)** bar chart of phylum-level relative abundance in ileum across groups; **(D)** bar chart of genus-level relative abundance in duodenum across groups; **(E)** bar chart of genus-level relative abundance in jejunum across groups; **(F)** bar chart of genus-level relative abundance in ileum across groups.

At the phylum level, the intestinal microbiota of the same intestinal segment in bullfrogs at different developmental stages was analyzed, and the species sequencing results were obtained (Figure 7). Within the intestinal microbiota of the duodenum, jejunum, and ileum in bullfrogs, Firmicutes, Proteobacteria, Bacteroidetes, Fusobacteria, and Actinobacteria represented the five phyla with relatively high abundances, collectively accounting for over 80% of the total sequences. This highlights the dominant role of these phyla in the intestinal microecology of bullfrogs.

The distribution characteristics of bacterial genera in the duodenum, jejunum, and ileum of bullfrogs across different developmental stages were analyzed at the genus level (Figure 7). In the duodenum, *Weissella* and *Lactococcus* exhibited higher abundances in Groups F and G, with the lowest levels observed in Group E; *Prevotella* reached 27% in Group E; *Clostridium* and *Corynebacterium* were more abundant in Group D, whereas their abundances in other groups were only 0.02–0.7%. In the jejunum, *Weissella* and *Lactococcus* were significantly more abundant in Groups F and G than in the other two groups, with Group E showing the lowest levels by contrast; *Prevotella* was the dominant genus in Group E, accounting for 49%, while its abundance was below 3% in other stages; *Leucobacter* displayed relatively high abundance in Group F. In the ileum, *Lactococcus* had a low abundance in Group E; the abundance of *Cetobacterium* gradually increased with post-metamorphic growth; *Prevotella* and *Phascolarctobacterium* were significantly more abundant in Group E than in other groups.

**Figure 8:**
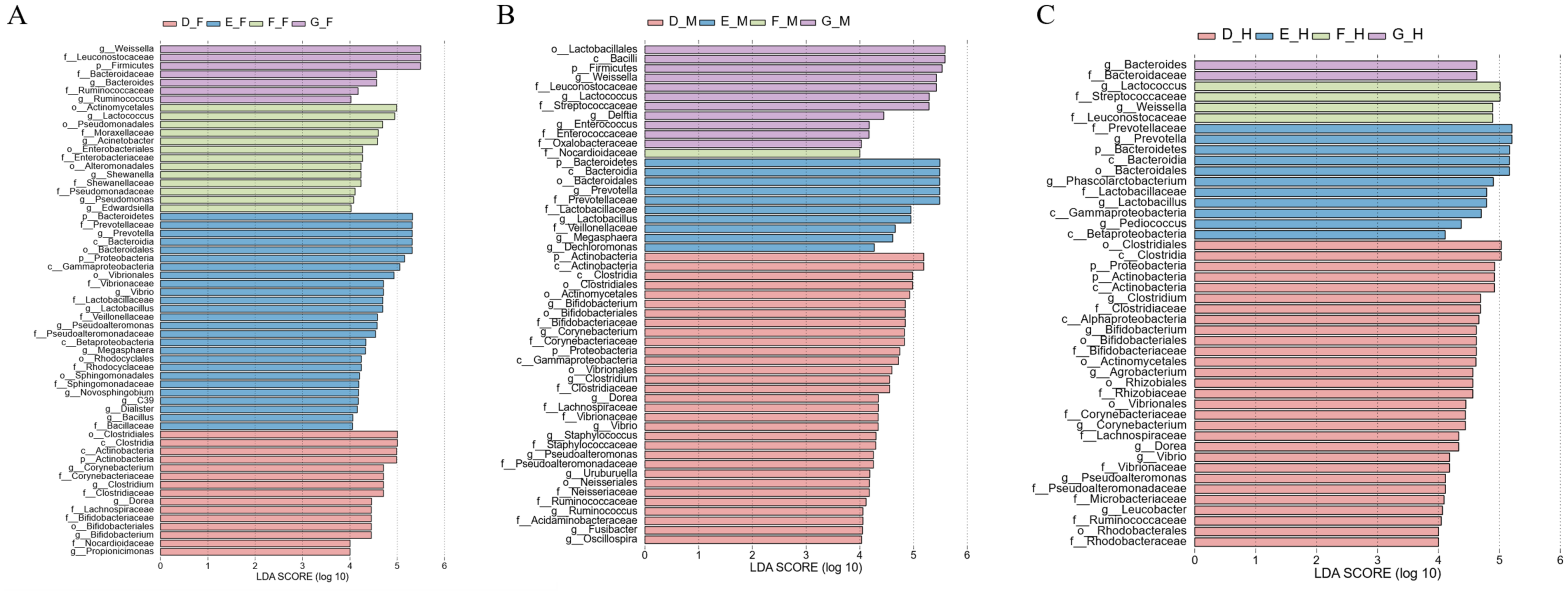
The LDA histogram of microbial genus level in the same intestinal segment of bullfrog at different stages was analyzed by LEfSe, with LDA > 4. **(A)** Duodenum across groups; **(B)** jejunum across groups; **(C)** ileum across groups.

Sixty-one significantly different bacterial taxa were identified in the duodenum (Figure 8, *p* < 0.05), with Group E harboring the highest number of enriched taxa (25 in total), including *Prevotella*, *Vibrio*, and *Lactobacillus*. In the jejunum, 53 bacterial taxa showed significant differences (*p* < 0.05), among which Group D was the most distinct, with 31 genera significantly enriched, such as *Bifidobacterium*, *Corynebacterium*, and *Clostridium*. Forty-six significantly different bacterial taxa were identified in the ileum (*p* < 0.05), with 29 taxa significantly enriched in Group D; representative genera included *Clostridium*, *Bifidobacterium*, and *Agrobacterium*.

**Figure 9:**
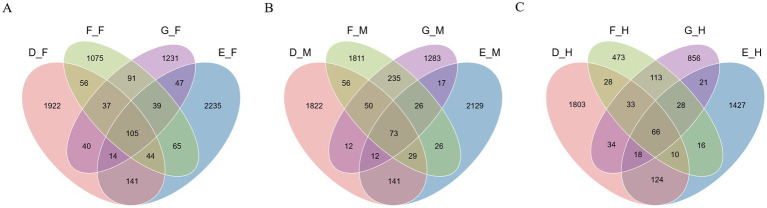
OTUs microbe in the same intestinal segment of bullfrog at different stages. **(A)** Duodenum across groups; **(B)** jejunum across groups; **(C)** ileum across groups.

As further indicated (Figure 9), the total number of OTUs across all samples from the duodenum, jejunum, and ileum was 7,142, 7,722, and 5,050, respectively. Along the intestinal tract from proximal to distal segments (i.e., from duodenum → jejunum → ileum), the number of shared OTUs across different age groups within the same segment decreased. Within the duodenum, jejunum, and ileum, Groups F and G exhibited the highest number of shared OTUs, with 272, 384, and 240, respectively.

Overall, analysis of bullfrogs’ intestinal microbiota across different developmental stages and segments (duodenum, jejunum, ileum) showed four key findings: five phyla (Firmicutes, Proteobacteria, Bacteroidetes, Fusobacteria, Actinobacteria) dominated the community (>80% of total sequences) at the phylum level; genus-level abundances varied by stage and segment (e.g., Weissella/Lactococcus higher in Groups F/G, Prevotella higher in Group E); significantly differential taxa were most enriched in Group E (duodenum, 25 taxa) and Group D (jejunum/ileum, 31/29 taxa); and shared OTUs across ages in the same segment decreased, with Groups F/G having the highest shared OTUs in each segment.

#### 3.4.2 Growth stage-dependent changes in microbial diversity in bullfrog jejunum and ileum

In the jejunum, the Shannon index decreased with advancing growth stage, with significant differences between Groups D/E and Groups F/G (*p* < 0.05). In the ileum, the Simpson index decreased as growth stages advanced, and there were significant differences between Group D and Groups F, G (*p* < 0.05) (Figure 3).

#### 3.4.3 Microbial community structure in bullfrog intestinal segments varies by growth stage

**Figure 10:**
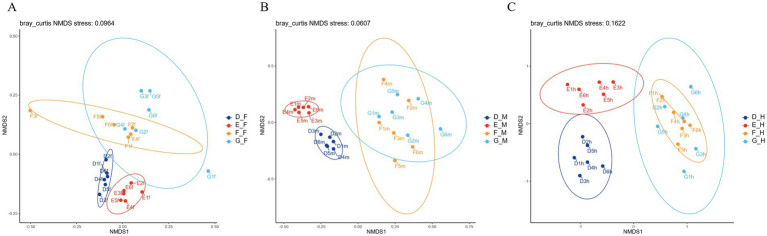
NMDS analysis of bullfrog microbes in the same intestinal segment at different stages: **(A)** Duodenum across groups, **(B)** jejunum across groups, **(C)** ileum across groups.

Results of microbial beta diversity analysis in the same intestinal segment of bullfrogs across different stages (Figure 10) showed that the bacterial community structures among different stage groups in the duodenum showed no obvious separation. In the jejunum, the intestinal microbiota of Groups D and E were significantly separated in clustering from that of Groups F and G, while Groups F and G exhibited high compositional similarity. In the ileum, the intestinal microbiota of Groups F and G overlapped to a large extent.

### 3.5 Core microbiota and stage-specific bacterial taxa in bullfrog intestinal development

**Figure 11:**
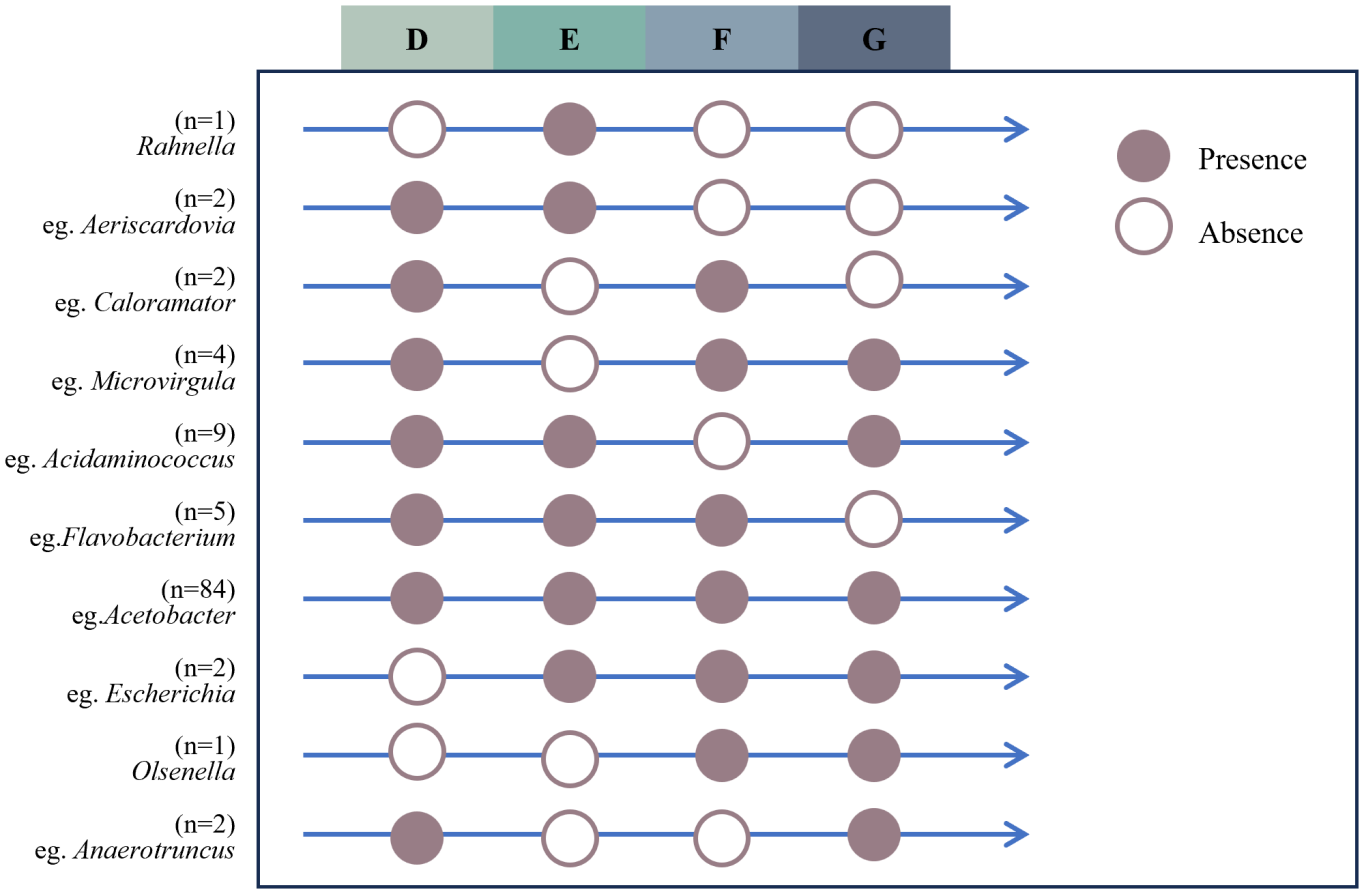
Longitudinal patterns of intestinal microbiota of bullfrog at different stages. Purple circles indicate the presence of microbial genera, while white circles indicate the absence.

A dataset was constructed by extracting bacteria shared bacterial taxa across the four developmental stages, and the longitudinal developmental pattern of the bullfrog intestinal microbiota was visualized (Figure 11). The results indicated that one genus was unique to Group E, namely *Rahnella*; 84 microbial species were present across all stage groups, including *Acetobacter* and *Mitsuokella*.

## 4 Discussion

Following morphological transformation, bullfrogs undergo functional remodeling of the intestinal system, where the co-evolution of intestinal morphological parameters and microbial community structure plays a key regulatory role in their growth and development. Intestinal morphological parameters—including villus height and width, as well as intestinal muscular layer thickness—serve as critical indicators for assessing intestinal development, health status, and digestive-absorptive capacity (Zheng et al., 2023). The findings of this study reveal that the rapid post-metamorphic body weight gain in bullfrogs is significantly associated with the adaptive development of intestinal morphology. With increasing age (in months), the continuous elongation of intestinal villi directly supports the energy requirements for weight gain by expanding the absorptive surface area and enhancing nutrient uptake efficiency (Fonseca-García et al., 2017). The small intestinal epithelium comprises a single layer of columnar cells, overlaid with numerous intestinal villi that are themselves covered by microvilli; this multi-tiered structure markedly increases the intestinal absorptive surface area (Fedi et al., 2021). During the period of rapid growth (from the second to the third month post-metamorphosis), the thickness of the intestinal muscular layer increases significantly. Research indicates that such muscular layer thickening directly reflects the adaptive demand for enhanced intestinal peristaltic capacity (Jing et al., 2024). This coordinated development of intestinal villi and muscular layer optimizes digestive and absorptive efficiency, creating a microenvironmental foundation for the stage-specific succession of intestinal microbiota. In summary, post-metamorphic intestinal morphological remodeling in bullfrogs provides structural support for host growth, while the synchronized shift in microbial communities further complements this functional optimization.

The present study identified Firmicutes, Proteobacteria, Bacteroidetes, Fusobacteria, and Actinobacteria as the dominant phyla in the intestinal microbiota of bullfrogs, a finding consistent with previous investigations (Kohl et al., 2013; Li et al., 2008). Jiang et al. (2022) noted that Firmicutes, Bacteroidetes, and Proteobacteria are likely indigenous to the intestinal tracts of frogs. The intestinal microbiota of bullfrogs exhibits distinct dynamic succession at the genus level across different post-metamorphic developmental stages: *Lactococcus* and *Bifidobacterium* predominate at 1 month of age, followed by a shift to *Prevotella* and *Phascolarctobacterium* at 2 months, with *Weissella* and *Bacteroides* gradually becoming dominant from 3 to 4 months. This stage-specific succession is not random but closely aligned with the shifting physiological demands of the host (e.g., immune maturation, energy metabolism optimization, and digestive function enhancement) during development. Notably, the genus-level microbial succession precisely mirrors the host’s developmental transition from immune vulnerability and high energy demand in early stages to efficient nutrient utilization and stable physiological function in later stages.

At 2 months of age, *Bifidobacterium* contributes to intestinal barrier formation through immunomodulatory activities (Lim and Shin, 2020), while *Lactococcus* exerts its function through primary metabolic processes (Wang et al., 2024). *Bifidobacterium* enhances immune tolerance by upregulating regulatory T cells (Tregs), strengthens intestinal barrier integrity, and maintains host immune homeostasis by inhibiting proinflammatory Th17/Th2 responses through short-chain fatty acids (e.g., butyrate) (Gavzy et al., 2023). Additionally, *Bifidobacterium animalis subsp. Lactis* improves host resistance to pathogens by activating intestinal Th1/Th17 cells and Tregs in neonates, thereby enhancing antimicrobial barrier function and suppressing *Salmonella* infection (Lin et al., 2024). *Lactococcus* utilizes lactose as its core carbon source, rapidly generating lactic acid through glycolysis to supply energy to the host, reduce intestinal pH, and inhibit pathogenic colonization. Its metabolic flexibility further ensures stable energy provision and intestinal microenvironmental homeostasis (Kleerebezem et al., 2020). As intestinal probiotics, *Lactococcus* species exhibit multiple beneficial effects in aquaculture. Studies have demonstrated that *Lactococcus* can enhance the barrier function of the small intestinal mucosa, mitigate intestinal damage induced by harmful substances, and promote the proliferation and differentiation of intestinal epithelial cells, thus preserving the integrity of the intestinal mucosa (Saleena et al., 2023). Dietary supplementation of Lactococcus has also been shown to improve growth performance in aquaculture species (Paritova et al., 2024), further supporting its role in juvenile bullfrog growth. Collectively, Lactococcus and Bifidobacterium synergistically support the early post-metamorphic stage by balancing immune protection and energy supply, adapting to the host’s immature physiological status.

By 3–4 months of age, the fiber-degrading capacities of *Prevotella* and *Phascolarctobacterium* contribute to enhanced nutrient absorption efficiency. *Prevotella* is associated with diets rich in plant-derived carbohydrates and fibers (Precup and Vodnar, 2019), and plays a pivotal role in fiber degradation in ruminants. Through synergistic interactions with other fiber-degrading bacteria, it facilitates the enzymatic hydrolysis of cellulose and hemicellulose (Zhang et al., 2020). Propionate, a metabolic byproduct of *Phascolarctobacterium* (Rainey et al., 2015), lowers intestinal pH, creating a favorable microenvironment that enhances the activity of fiber-degrading bacteria, thereby improving overall fiber decomposition capacity. In the later stages, the synergistic metabolism of proteins and polysaccharides by *Weissella* (Wang et al., 2014) and *Bacteroides* (Zafar and Saier, 2021) adapts to maximize energy supply required for rapid muscle growth. Notably, *Cetobacterium* persists as a core genus, with metabolic functions including the production of nutrients such as amino acids from various monosaccharides to support the host (Zhang H., 2023; Zhang Z. Y., 2023).

Genus-level analysis of bacterial communities across different intestinal segments of bullfrogs, *Weissella*, *Lactococcus*, *Cetobacterium*, *Prevotella*, *Lactobacillus*, and *Leucobacter* were identified as the dominant genera. Their distribution exhibited segment-specific patterns: the duodenum and jejunum were predominantly populated by *Weissella* and *Lactococcus*, while the ileum was dominated by *Cetobacterium* and *Lactococcus*. *Lactococcus* and *Weissella* can adapt to the microaerophilic environment of the anterior intestinal segments, inhibiting pathogenic colonization through the production of metabolic products such as lactic acid via fermentation (Khelissa et al., 2021; Teixeira et al., 2021). Certain strains of *Weissella* are capable of producing bacteriocins (e.g., Bacteriocins 7293A and 7293B), which exhibit strong inhibitory effects against Gram-negative pathogens including *Pseudomonas*, *Aeromonas*, *Salmonella*, and *Escherichia coli*, thereby preventing pathogenic colonization in the intestine (Singh et al., 2024). The strictly anaerobic nature of *Cetobacterium* enables its survival in the ileum, relying on the mucosal layer and fibrous substances not fully degraded by the anterior intestinal segments (Zhang et al., 2023). Through degrading complex polysaccharides, maintaining intestinal homeostasis, and optimizing energy acquisition, these bacterial communities support the adaptive growth of bullfrogs in aquaculture environments.

The present study revealed a decreasing trend in the Simpson index during bullfrog development, indicating a more uniform species distribution and a corresponding increase in microbial richness within the intestinal microbiota. This finding aligns with Yang Mengxiao’s research on *Pelophylax nigromaculatus* (Yang, 2023). Similarly, the intestinal microbiota of the Chinese giant salamander (*Andrias davidianus*) exhibits increasing complexity in both abundance and diversity with age, presumably linked to the expansion of dietary diversity. Higher microbial diversity and abundance are believed to facilitate the digestion of complex foods and efficient nutrient absorption in the host (Zhang, 2018). NMDS analysis based on Bray-Curtis distances demonstrated that the intestinal microbiota composition in the duodenum, jejunum, and ileum of bullfrogs at the same developmental stage was comparable. This similarity may arise from the structural and functional proximity of these interconnected intestinal segments, which promote microbial transmission and colonization across sites, thereby shaping convergent community compositions. Furthermore, developmental stage and feeding habits are likely to exert more pronounced effects on the beta diversity of intestinal microbiota (Shi et al., 2023). In this study, significant differences in intestinal microbiota composition across segments were observed between 2-month-old and 3-month-old bullfrogs, whereas 3-month-old and 4-month-old individuals displayed high inter-segment similarity—consistent with findings in the concave-eared frog (*Odorrana tormota*) (Shi et al., 2023). Juvenile frogs, in the early stages of development, exhibit immature intestinal and organ systems with relatively weak digestive and absorptive capacities (Zeng, 2023). In contrast, adult frogs possess well-developed intestinal structures and functions, enabling more efficient digestion and nutrient uptake. These physiological differences induce variations in the intestinal microenvironment, which in turn modulate the composition and structure of the intestinal microbiota.

Notably, while the present study systematically uncovers the associations between intestinal morphology, microbial community dynamics, and host growth, it has certain limitations that should be acknowledged. This study only establishes correlations through “morphological observations (intestinal villi, muscular layer) and microbial community structure analysis (16S rRNA sequencing)” without conducting functional validation, which results in the conclusion of “microbes regulating host physiology” remaining at the speculative stage with two key evidence gaps. Firstly, critical intestinal functional indicators were not detected, making it impossible to quantify the actual impact of microbes on host physiology. Secondly, *in vitro* validation or intervention experiments are lacking, which hinders the confirmation of the causal relationship between microbes and host physiological traits. The observed correlations could potentially be driven by third-party factors such as host endocrine changes during development, rather than direct microbial regulation. These limitations highlight the need for future studies to integrate functional assays and intervention experiments to establish a more robust causal link between intestinal microbiota and host physiology in bullfrogs.

## 5 Conclusion

Analyzing growth performance, intestinal morphology, and microbiota dynamics of 1–4-month-old post-metamorphic bullfrogs, this study found: Bullfrog growth rate (notably at 2–3 months) was significantly positively correlated with intestinal morphological development (villus height, muscular layer thickness). The microbiota was dominated by Firmicutes and Proteobacteria; dominant genera shifted with stages from *Bifidobacterium* and *Cetobacterium* (1–2 months, focusing on immunoregulation and basal metabolism) to *Weissella* and *Lactococcus* (3–4 months, enabling efficient energy conversion). Microbial diversity decreased with age; intestinal segment-specific microbiota exhibited significant differences at 2–3 months. This study clarifies the dynamic adaptation of microbiota to host growth needs, providing a basis for screening stage-specific probiotics for bullfrogs.

## Data Availability

The original contributions presented in the study are included in the article/Supplementary material, further inquiries can be directed to the corresponding authors.
